# Correlation of Severity of COVID-19 Disease With Gastrointestinal Manifestations and Liver Injury - A North Brooklyn Community Hospital Experience: A Retrospective Cohort Study

**DOI:** 10.7759/cureus.14543

**Published:** 2021-04-18

**Authors:** Kitson Deane, Ajay Singh, Azza Sarfraz, Zouina Sarfraz, Lyam Ciccone, Beishi Zheng, Arslan Afzal, Gulam Khan, Giovanna Rodriguez, Gul Bahtiyar

**Affiliations:** 1 Internal Medicine, Woodhull Medical Center, Brooklyn, USA; 2 Internal Medicine, Metropolitan Hospital, New York City, USA; 3 Pediatrics, Aga Khan University, Karachi, PAK; 4 Research and Academic Affairs, Fatima Jinnah Medical University, Lahore, PAK; 5 Gastroenterology, Woodhull Medical Center, Brooklyn, USA; 6 Endocrinology, Woodhull Medical Center, Brooklyn, USA

**Keywords:** gastrointestinal, symptoms, liver, transaminases, prognosis, covid-19

## Abstract

Introduction

The primary receptor for SARS-CoV-2 infection, angiotensin-converting enzyme-2 (ACE-2), is expressed in the gastrointestinal tract and liver parenchyma. The involvement of the gastrointestinal tract with severe acute respiratory syndrome coronavirus-2 (SARS-CoV-2) infection has remained unclear. The following study retrospectively reviews gastrointestinal symptoms and liver function tests at the time of hospital admission to identify patient outcomes including prolonged hospital stay, the requirement for intensive care, and all-cause in-hospital 30-day mortality.

Methods

A retrospective review of patient charts at the Woodhull Medical and Mental Health Center (WMC) was conducted at the time of hospital admission, using a pre-determined selection criterion. All adult patients, both inpatient and outpatient, were included from March 2020 till May 2020. A 95% confidence interval was used to estimate the odds ratio (OR) for patient outcomes.

Results

Of the 520 patients, gastrointestinal symptoms including nausea (OR = 0.375, p = 0.015), and nausea and vomiting in combination (OR = 0.400, p = 0.016) had an inverse protective relationship with all-cause in-hospital 30-day mortality among COVID-19 patients. Gastrointestinal symptoms including diarrhea (OR = 1.008, p < 0.001), and nausea and vomiting (OR = 1.291, p = 0.043) had a mild impact on the length of hospital stay.

Conclusion

Elevated liver transaminases including alanine transaminase (ALT) and aspartate transaminase (AST) at the time of hospital admission can predict critical care requirement and all-cause 30-day hospital mortality in patients with COVID-19 infection. Presence of gastrointestinal symptoms is associated with worsened outcomes.

## Introduction

Ever since coronavirus disease 2019 (COVID-19) was declared a global health emergency, it has caused over 2.48 million deaths as of February 23, 2021. The respiratory tract manifestations have been commonly observed with fever and cough being reported most frequently [1]. However, recent studies have demonstrated that the gastrointestinal tract and liver may also serve as target organs for severe acute respiratory syndrome coronavirus-2 (SARS-CoV-2) [2]. Such findings are corroborated on the basis that the angiotensin-converting enzyme 2 (ACE-2), the primary receptor for SARS-CoV-2, is presented in the gastrointestinal tract and liver parenchyma [3]. While the impact of SARS-CoV-2 on the gastrointestinal tract and liver remains unclear, COVID-19 patients presenting with digestive symptoms and deranged liver function tests (LFTs) may have a worse prognosis [4].

We conducted a retrospective review of patient data to correlate the presence of digestive symptoms and derangement of LFTs with the duration of hospital stay, the requirement for intensive care, and 30-day mortality of COVID-19 patients admitted to Woodhull Medical Center from March 2020 till May 2020.

## Materials and methods

Study design and population

A retrospective review of patient charts at the Woodhull Medical and Mental Health Center (WMC) was conducted. All patients aged 18 years and above were included with no restriction to gender, race, or ethnicity. Only pre-existing data, both inpatient and outpatient, was included from March 2020 till May 2020. Data were obtained from patient records, both electronic and paper records, from the WMC. Specific data were reviewed including patient demographics, gastrointestinal symptomatology, laboratory values at the time of hospital admission, progress notes, and procedure notes.

Data collection

Privacy and security were maintained by password protecting identifiable data and minimizing access to identifiable data. All datasheets were password protected and all paper records were stored in a keyed file drawer in a locked office. Following the acquisition of information, surrogate numbers were given to identify patients for data analysis, and a key was kept in a separate password-protected excel sheet to be searched until after completion of the study at which time it was deleted. The principal and co-investigators reviewed the charts and were the only members to have access to the subjects' data.

Statistical analysis

The statistical analysis was conducted to assess the role of various factors in patient outcomes. The frequency of gastrointestinal symptoms and elevated LFTs were specified. Only the parameters obtained at the time of hospital admission were used to conduct the statistical analysis. The primary outcome was to determine the 30-day mortality, ICU admission, and hospital stay following COVID-19 diagnosis for enrolled patients. The results of the multivariate analysis were presented in terms of the odds ratio (OR). A difference with a two-sided α < 0.05 was considered statistically significant. The data analysis was conducted on Statistical Package for Social Sciences (SPSS) version 26.0 (IBM Corp., Armonk, NY).

## Results

A total of 520 patients were reviewed retrospectively who were polymerase chain reactive (PCR) positive for COVID-19. Among these, 60 patients had only nausea, 37 patients had only vomiting, and 63 patients with nausea and vomiting. Only 64 patients had diarrhea as their only gastrointestinal manifestation. A total of 105 patients had a combination of one or more of nausea, vomiting, or diarrhea (Table 1).

 

**Table 1:** Gastrointestinal symptomatology of participants

	All patients	Patients with nausea	Patients with vomiting	Patients with nausea and vomiting	Patients with diarrhea	Patients with any combination of one or more of the 3
n	520	60	37	63	64	105

Of the 520 patients, 344 patients had deranged international normalized ratio (INR) (Table 2). Nearly all of the patients (n = 488) had elevated aspartate transaminase (AST) and alanine transaminase (ALT) in combination (Table 2).

**Table 2:** Laboratory findings of participants INR: International normalized ratio; AST: Aspartate transaminase; ALT: Alanine transaminase.

	All patients	Patients with deranged INR	Patients with elevated AST and ALT	Patients with decreased albumin (<3.5)
n	520	344	488	481

Patients presenting with nausea (OR = 0.375, p = 0.015), as well as nausea and vomiting in combination (OR = 0.400, p = 0.016), had a significant 30-day mortality rate (Table 3). Elevation of ALT (OR = 1.558, p = 0.038), AST (OR = 2.743, p < 0.001), and in combination (OR = 2.593, p < 0.001) had a significant impact on the 30-day mortality rate (Table 3).

**Table 3:** Predictors of 30-day mortality rate INR: International normalized ratio; AST: Aspartate transaminase; ALT: Alanine transaminase.

Symptoms	30-day mortality rate	Odds ratio (OR)	95% Confidence Interval				P-value
				Lower	Upper		
Nausea	5.8%	0.375	0.166		0.849		0.015
Vomiting	4.2%	0.471	0.179		1.239		0.160
Diarrhea	9.0%	0.630	0.325		1.22		0.222
Nausea and Vomiting	6.7%	0.400	0.185		0.864		0.016
Elevated INR (>1.5)	17.3%	1.824	0.941		3.536		0.072
Elevated Transaminases (AST > 40 and ALT > 45)	78.2%	2.593	1.635		4.111		0.000
AST > 40	33.6%	2.743	1.739		4.327		0.000
ALT > 45	32.9%	1.558	1.023		2.372		0.038
Low Albumin (<3.5)	43.9%	1.120	0.749		1.676		0.580

Diarrhea as a gastrointestinal (GI) manifestation was a significant contributor to prolonged hospital stay lasting ≥ 10 days (OR = 1.008, p < 0.001) (Table 4). Nausea and vomiting were also associated with prolonged hospital stay lasting ≥10 days (OR = 1.291, p = 0.043) (Table 4).

**Table 4:** Predictor of prolonged hospital stay (≥10 days) INR: International normalized ratio; AST: Aspartate transaminase; ALT: Alanine transaminase.

Symptoms	Hospital Stay		OR	95% Confidence Interval		P-value
	<10 days	≥10 days		Lower	Upper	
Nausea	11.0%	13.9%	1.307	0.760	2.249	0.332
Vomiting	7.2%	8.0%	1.12	0.569	2.204	0.742
Diarrhea	12.3%	12.4%	1.008	0.591	1.717	0.000
Nausea and Vomiting	12.0%	15.0%	1.291	0.764	2.182	0.043
Elevated INR (>1.5)	13.1%	11.1%	0.827	0.431	1.586	0.567
Elevated Transaminases (AST > 40 and ALT > 45)	64.3%	62.2%	0.915	0.633	1.323	0.637
AST > 40 only	61.5%	61.2%	0.989	0.686	1.424	0.952
ALT > 45 only	30.2%	29.2%	0.951	0.644	1.403	0.799
Low Albumin (<3.5)	39.7%	45.6%	1.275	0.887	1.832	0.188

Patients with elevated ALT only (OR = 1.594, p = 0.019), AST only (OR = 2.149, p < 0.001), and both ALT and AST (OR = 2.022, p < 0.001) were all significant predictors of intensive care unit (ICU)/progressive care unit (PCU) care (Table 5). Symptoms including nausea, vomiting, and diarrhea did not lead to increased odds of ICU/PCU care (Table 5).

**Table 5:** Predictor for requirement of ICU/PCU care ICU: Intensive care unit; PCU: Progressive care unit; INR: International normalized ratio; AST: Aspartate transaminase; ALT: Alanine transaminase.

Symptoms	ICU/PCU Care	OR	95% Confidence Interval		P-value
	Admission rates		Lower	Upper	
Nausea	10.1%	0.707	0.396	1.262	0.239
Vomiting	5.9%	0.712	0.34	1.491	0.366
Diarrhea	10.9%	0.827	0.475	1.442	0.504
Nausea and Vomiting	14.3%	0.761	0.435	1.331	0.337
Elevated INR (>1.5)	13.6%	1.206	0.636	2.286	0.566
Elevated Transaminases (AST > 40 and ALT > 45)	72.6%	2.022	1.37	2.986	0.000
AST > 40 only	71.6%	2.149	1.461	3.161	0.000
ALT > 45 only	36.0%	1.594	1.078	2.357	0.019
Low Albumin (<3.5)	46.2%	1.318	0.913	1.904	0.140

## Discussion

We conducted a retrospective review of patients admitted with gastrointestinal manifestations following COVID-19 infection. Gastrointestinal involvement was assessed by reviewing gastrointestinal symptoms, and liver function test abnormalities. Our findings demonstrate longer hospital stays (≥10 days) among patients manifesting with gastrointestinal symptoms including diarrhea, and a combination of nausea and vomiting. However, we also found that the presence of gastrointestinal symptoms was suggestive of a reduced 30-day mortality rate. Hepatic predictors including elevated ALT and AST had a strong association with the 30-day mortality rate and critical care requirement. Low albumin and elevated INR did not have any impact on the prognosis of patients with COVID-19 infection in our cohort.

The underlying mechanism for gastrointestinal involvement in COVID-19 is similar to that of respiratory and due to the attachment of the SARS-CoV-2 virus to the angiotensin-converting enzyme 2 (ACE-2) receptor in the digestive system, particularly the proximal large intestine [5]. Previous studies have reported data on the involvement of the gastrointestinal tract and liver [6,7]. Mao et al. identified a pooled prevalence of 15% for gastrointestinal symptoms in COVID-19 of whom 10% did not have respiratory involvement [2]. Gastrointestinal symptoms have been observed among patients with COVID-19 infection in literature with discrepancies in their predictive prognostic potential [8,9]. In our cohort, GI symptoms had an inverse protective relationship with the 30-day mortality rate. However, our analysis demonstrates a significant impact of diarrhea, and nausea, and vomiting in combination on the duration of hospital stay. Additionally, the gastrointestinal manifestations had no contribution to the patients' requirements for critical care admissions. With conflicting reports of the impact of gastrointestinal symptoms on COVID-19 infection outcomes, our analysis also demonstrates both their positive and negative prognostic potential.

Our findings suggest that, as the liver involvement is suggested through ALT and AST elevation, patients have worse 30-day mortality rates and require critical care with the difference proving significant. Reports of liver injury have been suggested earlier including elevated transferases and INR, and decreased albumin levels, among patients with COVID-19 [10,11]. While our findings did not identify low serum albumin as a prognostic marker as reported by Huang et al. [12], elevated transaminases were suggestive of worse outcomes in our cohort of COVID-19-infected patients. Our findings from recent literature show that elevated AST and/or ALT are associated with increased mortality rates.

Strengths and limitations

The main strengths of our study include a large sample size (n = 520) and the longitudinal study design in New York City, a prominent epicenter of the COVID-19 pandemic. The lab parameters and gastrointestinal manifestations were assessed on the day of admission. To the best of our knowledge, this is the first study that observes both liver function tests as well as gastrointestinal symptoms on the day of hospital admission to assess their prognostic potential.

The main limitations of our study include the lack of categorization of the existing burden of chronic liver disease and the use of medications that might be hepatotoxic. As the gastrointestinal symptoms and liver injury markers were assessed at the time of hospital admission, patients with pre-existing liver injury may have been included. No testing was conducted to assess the hepatitis B and C status of these patients. Similarly, the baseline comorbidities and other health parameters, i.e. presence of respiratory symptoms, were not controlled for in the analysis.

Recommendations

Gastrointestinal manifestations of COVID-19 may manifest early in the course of the disease, possibly occurring in isolation. Management of patients with COVID-19 infection should also contain the potential prognostic impact of liver markers and gastrointestinal symptoms. With a great interest in the prognostic capacity of gastrointestinal symptoms and liver involvement, this study identifies a worse prognosis among COVID-19 patients. While the main target organ of COVID-19 infection remains the respiratory system, the impact of gastrointestinal involvement may not be undermined as has been observed in the literature [13-15].

## Conclusions

Elevated liver transaminases including ALT and AST at the time of hospital admission can predict critical care requirement and all-cause 30-day hospital mortality in patients with COVID-19 infection. Presence of gastrointestinal symptoms is associated with worsened outcomes.
